# Stunting in hospitalized children: Prevalence and modifiable nutritional risk factors at a tertiary care hospital

**DOI:** 10.12669/pjms.42.5.13054

**Published:** 2026-05

**Authors:** Zia Muhammad, Muhammad Kashif

**Affiliations:** 1 Zia Muhammad, FCPS. Department of Child Health, Khyber Teaching Hospital, Peshawar, Pakistan; 2 Muhammad Kashif, FCPS. Department of Child Health, Khyber Teaching Hospital, Peshawar, Pakistan

**Keywords:** Breast Feeding, Child, Hospitalized, Dietary Supplements, Malnutrition, Nutritional Support, Risk Factors, Stunting

## Abstract

**Objectives:**

To determine the prevalence of stunting and identify its associated factors among hospitalized children

**Methodology:**

This descriptive cross-sectional single center study was carried out at Peads “B” Unit Khyber Teaching Hospital Peshawar from February 2020 to December 2024. A sample of 760 children between six and 60 months admitted in medical pediatric wards was enrolled in the study. Structured caregiver interviews, review of hospital records and standardized anthropometry measurements were used to collect data. Height-for-age z-scores were calculated using WHO Anthro software. Logistic regression was used to identify independent predictors of stunting.

**Results:**

Among 760 hospitalized children, the mean age was 28.3 ± 13.7 months, and 56.8% were male. The prevalence of stunting was 35.9% (95%CI: 32.5%–39.3%), including 24.7% with moderate stunting and 11.2% with severe stunting. Independent risk factors for stunting included exclusive breastfeeding for less than six months (AOR = 1.84, 95%CI: 1.32–2.55; p = 0.001), untimely complementary feeding (AOR = 1.98, 95%CI: 1.45–2.72; p < 0.001), no zinc supplementation (AOR = 1.67, 95%CI: 1.19–2.34; p = 0.003), feeding difficulties (AOR = 2.31, 95%CI: 1.62–3.30; p < 0.001), hospital stay of more than seven days (AOR = 1.74, 95%CI: 1.24–2.42; p = 0.001), and no feeding support during hospitalization (AOR = 1.85, 95%CI: 1.34–2.55; p < 0.001).

**Conclusion:**

Stunting is still high among hospitalized children and is strongly associated with the modifiable nutritional and in-patient care practices. Routine feeding evaluation, intervention and parental education should be included in pediatric protocols for the prevention of this serious and often unrecognized complication.

## INTRODUCTION

Stunting is a sign of chronic undernutrition brought on by prolonged exposure to poor nutrition and infections during crucial stages of growth and development. It is defined by a height-for-age z-score of -2 standard deviations (SD) or more below WHO growth standards.^1^ In 2022, about 148.1 million children under five worldwide were affected by stunning, and it is a serious public health issue, particularly in low- and middle-income countries.^2^ Undernutrition has negative effects on cognitive development, academic performance, and future economic production in addition to its physiological effects.^3^,^4^

The proportion of stunting in children is more attributed to dietary’s factors, especially poor protein-energy intake and micronutrient deficiencies. Malnutrition usually starts in the in-utero period because of maternal undernutrition and progresses throughout infancy with suboptimal breast-feeding, inappropriate complementary feeding, and recurrent infections.^5^ Hospitalized children are especially vulnerable: anorexia, increased metabolic requirements, and extended hospitalizations can compound pre-existing nutritional deficiencies.^6^

In hospitals, where only severe forms of chronic malnutrition are recognized, stunting is often overlooked in clinical settings. Stunting is also more likely to occur in children who are hospitalized due to illness, but nutritional evaluation is not always performed.^7^ A worse-than-ideal recovery with a higher risk of complications and long-term developmental abnormalities might result from untreated stunting during hospitalization.^8^

In high-stunting regions such as South Asia and Sub-Saharan Africa with prevalence of stunting over 30%, the role of hospital practices is particularly important for early diagnosis and intervention.^9^ Nutritional assessment, personalized nutrition counseling, and micronutrient supplementation during hospitalization is an effective approach that can have significant effects.^10^ However, there is little information about how well hospitals are able to manage chronic undernutrition in hospitalized pediatric populations.

Besides that, the COVID-19 pandemic has perhaps caused a change in the number of pediatric hospitalizations and the provision of nutritional services. Delayed care seeking, interference with regular child health care, less access to counseling, and lack of nutritional assessment and support can have led to the high susceptibility of hospitalized children to malnutrition as well as may have contributed to admission patterns. This background also justifies the importance of assessing cases of stunting and associated nutritional risk factors in hospitalized children.

It is an under-investigated area despite national and international policies targeting reduction of stunting. Despite the significance of national health survey data related to predictors of stunting in the community, the data fails to capture the problems related to the hospital setting including feeding challenges due to illness, hospital prolongations and lapses in inpatient nutritional care. These factors could be used to gain insight and develop specific hospital-based interventions to mitigate paediatric stunting. This study was therefore carried out to determine the burden of stunting in hospitalized children and the associated nutritional predictors with the view to propose hospital-based protocols for the early detection and management of chronic malnutrition.

## METHODOLOGY

This descriptive cross-sectional study was carried out in the Pediatric Medical “B” Unit of Khyber Teacing Hospital, Peshawar, a major teaching hospital and referral center for tertiary care in Khyber Pakhtunkhwa, Pakistan. Data collection was done over five years (February 2020 to December 2024. A non-probability consecutive sampling technique was employed, where all eligible six months to five years-old children admitted to Paediatric Medical Unit B during the study period were screened for stunting and if they met the inclusion criteria, enrolled in the study.

### Inclusion and Exclusion Criteria:

In total, 760 paediatric inpatients were included. The sample size of 760 was given based on the consecutive enrolment of all the eligible children who were available to be enrolled in the study at any given time and not a given sample size made by a formal sample size calculator. Children with congenital anomalies, chromosomal anomaly like Down syndrome or chronic illnesses which are known to influence nutritional status or linear growth were excluded.

Chronic illness was considered as a long-term medical condition previously diagnosed as such which has to continue to be followed up or treated and which is anticipated to influence the nutritional status or linear growth. This was a case of thalassemia major, malignancy, chronic kidney disease, chronic liver disease, cystic fibrosis, and congenital heart disease among others. Children whose anthropometric measurements or dietary data were not complete at the time of measurement and those who were readmitted were also excluded.

Prospective demographic, clinical, and nutritional data were gathered through a structured, pretested proforma through the course of hospitalization. The data was gathered by interviewing caregivers with a standardized questionnaire that included dietary history, and the feeding patterns. Hospital records and patient files were reviewed for clinical diagnosis and relevant clinical information. The anthropometric measurements were carried out with the help of trained nursing personnel by World Health Organization (WHO)-approved methods. Height and length (±0.1 cm) were measured with the help of an infantometer/stadiometer, and weight (±100 g) was measured with the help of a digital pediatric scale. The weight-for-age and height-for-age z-score were calculated using WHO Anthro software version 3.2.2. According to the WHO Child Growth Standards, stunting was defined as a height-for-age z-score (HAZ) of < -2 standard deviations.

Caregiver interview and clinical records review were used to evaluate several nutritional and hospital-related variables. History of dietary habits comprised of breastfeeding status, length of exclusive breastfeeding, and age at which complementary feeding started. Timely complementary feeding was said to be one with the initiation at the age of six months, whereas untimely complementary feeding was that of initiating before or after the age of six months. Records of information on the micronutrient supplementation such as iron, zinc and multivitamins were also recorded depending on the caregiver report. Feeding issues such as inadequate appetite, refusal to eat, vomiting, and diarrhea were examined at the admission point as per the reported symptoms in the recent past according to the caregiver and the clinical assessment. Hospital-related variables were length of stay, infection at admission, and feeding assistance during hospitalization. Feeding support was defined as nutritional support offered at the time of admission. Caregiver report on monthly family income was also collected using structured proforma and the data was categorized as <PKR 25,000 and ≥PKR 25,000 for analysis. To conduct multivariable analysis, certain variables were recoded into binary composite variables, such as no zinc supplementation (based on zinc supplementation status), feeding difficulties (any), which is defined as the presence of poor appetite, feeding refusal, vomiting, or diarrhea, and lack of feeding support during hospitalization (based on feeding support provided during hospitalization).

### Ethical Approval:

The Institutional Review Board (IRB) of Khyber Teaching Hospital Peshawar granted ethical approval for the study (Ref# 763/ADR/KMC, dated February 10, 2020).

### Statistical Analysis:

The data were entered and analyzed with IBM SPSS 25.0. The baseline characteristics were summarized using descriptive statistics (means ± standard deviations for continuous variables and frequencies and percentages for categorical variables). The stunting prevalence was computed, and the bivariate analysis was done using the chi-square test to determine the relationship between stunting and the clinical and nutritional variables. Significant variables identified in the bivariate analysis process were added to multivariate logistic regression to determine the independent relationship between different factors and stunting. Odds ratios (AORs) with 95% confidence intervals were presented. A p-value of ≤ 0.05 was considered statistically significant.

## RESULTS

The mean age was 28.3 ± 13.7 months, with 56.8% males. The highest proportion of admissions came from low-income families and rural areas Table-I. Out of 760 children, 273 (35.9%) were stunted, including 188 (24.7%) with moderate stunting and 85 (11.2%) with severe stunting Table-II.

**Table-I:** Baseline sociodemographic characteristics of the study population (n = 760).

Variable	Frequency (n)	Percentage (%)
Age (mean ± SD)	28.3 ± 13.7	-
***Gender***		
Male	432	56.8
Female	328	43.2
***Residence***		
Urban	301	39.6
Rural	459	60.4
***Monthly Income***		
< 25,000 PKR	512	67.4
≥ 25,000 PKR	248	32.6

**Table-II:** Prevalence of Stunting.

Category	Frequency (n)	Percentage (%)
Not Stunted	487	64.1
Moderately Stunted	188	24.7
Severely Stunted	85	11.2
Total Stunted	273	35.9

Table-III shows the feeding practices in the study population across growth status (with stunting). Eighty-two-point five percent children were ever breastfed, 44.1% were exclusively breastfed for six months and 57.8% received timely complementary feeding in the overall subgroup of children (Table-III). Exclusive or mixed breastfeeding and untimely complementary feeding were observed to be significantly related stunting stages; however no significant association was seen for ever breastfeeding.

**Table-III:** Feeding practices and their association with stunting.

Feeding Practice	Total n (%)	Stunted n (%)	Not Stunted n (%)	p-value
Ever breastfed	627 (82.5)	226 (82.8)	401 (82.4)	0.289
Exclusive breastfeeding (6 months)	335 (44.1)	95 (34.8)	240 (49.3)	0.001
Timely complementary feeding (at 6 months)	439 (57.8)	120 (44.0)	319 (65.5)	<0.001

The correlations among stunting and nutritional, feeding and hospital related variables are indicated in Table-IV. Stunted children were less likely to report zinc supplementation as compared to non-stunted children (66/273, 24.2% vs. 176/487, 36.1%; p = 0.001). Other differences that were observed in the stunted group were high rates of poor appetite (129/273, 47.3% vs. 129/487, 26.5%), vomiting/diarrhea (78/273, 28.6% vs. 86/487, 17.7%), and feeding refusal (64/273, 23.4% vs. 58/487, 11.9%). Moreover, longer than seven days stay in hospital, being infected at hospitalization, and no feeding assistance during hospitalization was much more prevalent among stunted children compared to non-stunted children (p < 0.001) (Table-IV).

**Table-IV:** Association of nutritional, feeding, and hospital-related factors with stunting status.

Variable Category	Specific Variable	Stunted (n=273)	Not Stunted (n=487)	p-value
Micronutrient Use	Iron supplementation	98 (35.9%)	212 (43.5%)	0.04
	Zinc supplementation	66 (24.2%)	176 (36.1%)	0.001
	Multivitamin use	109 (39.9%)	248 (50.9%)	0.003
Feeding Difficulties	Poor appetite	129 (47.3%)	129 (26.5%)	<0.001
	Vomiting/diarrhea	78 (28.6%)	86 (17.7%)	0.001
	Feeding refusal	64 (23.4%)	58 (11.9%)	<0.001
Hospital-related Factors	Length of stay > 7 days	121 (44.3%)	132 (27.1%)	<0.001
	Infection at admission	193 (70.7%)	277 (56.9%)	<0.001
	Feeding support provided	98 (35.9%)	264 (54.2%)	<0.001

Significant variables in bivariate analysis were then included in multivariate logistic regression in order to determine independent predictors of stunting. Less than six months of exclusive breastfeeding, late complementary feeding, and the lack of zinc supplement, feeding problems, hospital stay longer than seven days, and feeding assistance during hospitalization were found to be independently correlated with stunting (Table-V).

**Table-V:** Multivariate Logistic Regression for Predictors of Stunting.

Variable	AOR	95% CI	p-value
Exclusive Breastfeeding < 6 months	1.84	1.32-2.55	0.001
Untimely Complementary Feeding	1.98	1.45-2.72	<0.001
No Zinc Supplementation	1.67	1.19-2.34	0.003
Feeding Difficulties (any)	2.31	1.62-3.30	<0.001
Hospital Stay > 7 days	1.74	1.24-2.42	0.001
No feeding support during hospitalization	1.85	1.34-2.55	<0.001

## DISCUSSION

This study identified a high burden of stunting (35.9%) among hospitalized children aged 6-59 months in Khyber Teaching Hospital, Peshawar, thereby, emphasizing chronic undernutrition as a common problem even at tertiary care hospitals. This proportion is similar to the national estimates as shown in Pakistan Demographic and Health Survey (PDHS, 2018)^12^ which revealed stunting in up to 37.6% of children under five years, indicating low nutritional status as a major public health concern despite the availability of hospital care.^11^

In this study, suboptimal feeding practices were highly related to stunting, but as a result of the cross-sectional nature, the results are best viewed as associations, and not temporal or causal relationships. The odds of stunting were nearly doubled for children who were not exclusively breastfed for the first six months (AOR = 1.84, p = 0.001), which is in line with the findings of Black et al.,^13^ who found that exclusive breastfeeding significantly lowers the risk of stunting by shielding children from early infection exposure and low-quality complementary foods. Early or late onset of complementary feeding was also a significant predictor (AOR = 1.98, p < 0.001), which is in line with Dewey et al. finding that improper timing of complementary feeding disrupts normal growth trajectories.^14^

These findings highlight the need to strengthen WHO feeding recommendations in clinical practice at the time of hospitalization, particularly in high-risk pediatric sector. Even though breastfeeding rates in this population were high (> 80%), the consistency and quality of breastfeeding practices provided inadequate protection against growth faltering.

Another independent risk factor was not taking zinc supplementation (AOR = 1.67, p = 0.003). This is in line with the findings of Bhutta et al.^14^, who emphasized zinc’s role in enhancing immune function, improving appetite, and supporting linear growth. Similarly, children who were stunted were more likely to not take multivitamins or iron supplements, but this was not included in the multivariate analysis; this could be because the dietary variables were redundant or because the dosage was not followed.

Poor appetite, feeding refusal or gastrointestinal symptoms were present in 47.3% of stunted children and were significantly associated with increased odds of stunting (AOR = 2.31 p < 0.001). This is in line with data in the literature indicating that disease-related anorexia, periodic diarrhea, and oral feeding difficulties cause nutritional decline.^15^ Furthermore, the high percentage of admission infections (70.7% among stunted children) reinforces the interplay between infection and undernutrition described in the UNICEF undernutrition conceptual framework.^16^ Interestingly, long hospital stays (>7 days), and lack of feeding support were significant independent predictors of stunting. Children hospitalized for over a week had 74% higher likelihood of stunting (AOR = 1.74, p = 0.001), indicating that persistent illness episodes and suboptimal inpatient care practices may accentuate pre-existing nutritional deficits in children. The absence of assist feeding plan in hospital stay was significantly associated with stunting (AOR = 1.85, p <0.001) which is consistent with Hossain et al.^17^ results, who emphasized the lack of nutrition care at the hospital level in low-income settings.

These results highlight a significant inadequacy in hospital nutrition policy and practice. Inpatient care tends to be acute-oriented, with little attention paid to routine nutritional screening, individualized feeding protocols or addressing chronic undernutrition during hospitalization. Similar findings were reported by studies in Bangladesh, Nigeria and India, where stunting among hospitalized children was 30%-45% and it was significantly associated with poor feeding practices, absence of supplements, and repeated infections.^18^-^20^ However, some variations exist. For instance, Chapagain et al.^21^ reported a small effect size between administering micronutrient supplementation and growth status, although this could be related to contrasting dosages, compliance or other factors like illness. In addition, the current study is novel in that the hospital environment (e.g., no feeding support) itself is identified as a modifiable risk factor, which has frequently been overlooked in previous community-based research.

The research possesses a number of strengths such as the application of WHO-standardized anthropometric measurements and a fairly large sample of hospitalized children. It also demonstrates significant nutritional and hospital-related factors that are related to stunting that can be used to inform inpatient nutritional assessment and support.

### Limitations:

The single-centered design can be a limitation to the generalizability. Also, there was a possibility of recall bias in caregiver-reported feeding practices. It is also needed to interpret the findings in the context of the potential impact of the COVID-19 pandemic that might have affected the pattern of hospital admissions, the behavior of people who use health care, and the access to nutritional assessment and support services. Lastly, due to the cross sectional design, causal relationships cannot be determined.

## CONCLUSION

Stunting remains predominant in hospitalized children and is associated with easily preventable nutritional and health factors such as suboptimal breastfeeding, delayed initiation of complementary feeding, micronutrient deficiencies, feeding difficulties, and inadequate in-hospital nutritional support. An integrated approach to nutrition care, routine screening, provision of micronutrients, and inpatient nutrition counselling is required for acute and chronic undernutrition in hospitals.

### Recommendations:

Multicenter prospective studies involving longitudinal follow-up should be carried out in the future to help identify temporality, causation, and additional applicability of the findings.

### Authors’ Contribution:

**ZM:** Study design & Concept, did statistical analysis & editing of manuscript responsible for integrity of research.

**MK:** Design of the study, Data collection, manuscript writing, review and final approval.

## References

[1] World Health Organization;United Nations Children's Fund (UNICEF);International Bank for Reconstruction and Development/The World Bank (2025). Levels and trends in child malnutrition:key findings of the 2025 edition of the Joint Child Malnutrition Estimates.

[2] Vaivada T, Akseer N, Akseer S, Somaskandan A, Stefopulos M, Bhutta ZA (2020). Stunting in childhood: an overview of global burden, trends, determinants, and drivers of decline. Am J Clin Nutr.

[3] Fatima S, Manzoor I, Joya AM, Arif S, Qayyum S (2020). Stunting and associated factors in children of less than five years: a hospital-based study. Pak J Med Sci.

[4] Joshi M, Uday S (2023). Vitamin D deficiency in chronic childhood disorders: importance of screening and prevention. Nutrients.

[5] Posam UM, Chereddy TC, Pirati RS, Rao NVR, Bandrapalli E, Yangalasetty S (2022). A cross-sectional study on assessment of nutritional status and factors affecting anemia in children in tertiary care teaching hospital. Int J Contemp Pediatr.

[6] Lee YM, Sohn MH, Kim J, Kim JH, Choe YH, Kim KM (2021). “Nationwide Pediatric Nutrition Day”survey on the nutritional status of hospitalized children in South Korea. Nutr Res Pract.

[7] Al-Waleedi AA, Al-Shammiry AA, Al-Shaibani AA (2022). Malnutrition among hospitalized children 12-59 months of age in Lahj and Abyan governorates, Yemen. BMC Nutr.

[8] Soofi SB, Khan A, Kureishy S, Hussain I, Habib MA, Umer M (2023). Determinants of stunting among children under five in Pakistan. Nutrients.

[9] Elia M (2001). The Malnutrition Advisory Group consensus guidelines for the detection and management of malnutrition in the community. Nutr Bull.

[10] Victora CG, Christian P, Vidaletti LP, Gatica-Domínguez G, Menon P, Black RE (2021). Revisiting maternal and child undernutrition in low-income and middle-income countries: variable progress towards an unfinished agenda. Lancet.

[11] World Health Organization (2006). WHO Child Growth Standards:length/height-for-age, weight-for-age, weight-for-length, weight-for-height and body mass index-for-age:methods and development.

[12] National Institute of Population Studies (NIPS) [Pakistan], ICF (2019). Pakistan Demographic and Health Survey 2017-18.

[13] Black RE, Victora CG, Walker SP, Bhutta ZA, Christian P, de Onis M (2013). Maternal and child undernutrition and overweight in low-income and middle-income countries. Lancet.

[14] Dewey KG, Adu-Afarwuah S (2008). Systematic review of the efficacy and effectiveness of complementary feeding interventions in developing countries. Matern Child Nutr.

[15] Bhutta ZA, Ahmed T, Black RE, Cousens S, Dewey K, Giugliani E (2008). What works?Interventions for maternal and child undernutrition and survival. Lancet.

[16] Ojofeitimi EO, Owolabi OO, Aderonmu A, Esimai AO, Olasanmi SO (2003). A study on under-five nutritional status and its determinants in a semi-rural community of Ile-Ife, Osun State, Nigeria. Nutr Health.

[17] Prendergast AJ, Humphrey JH (2014). The stunting syndrome in developing countries. Paediatr Int Child Health.

[18] Meshram II, Mallikharjun Rao K, Balakrishna N, Harikumar R, Arlappa N, Sreeramakrishna K (2012). Prevalence and determinants of undernutrition and its trends among pre-school tribal children of Maharashtra State, India. J Trop Pediatr.

[19] Islam MM, Sanin KI, Mahfuz M, Ahmed AMS, Mondal D, Haque R (2018). Risk factors of stunting among children living in an urban slum of Bangladesh: findings of a prospective cohort study. BMC Public Health.

[20] Akombi BJ, Agho KE, Hall JJ, Merom D, Astell-Burt T, Renzaho AMN (2017). Stunting and severe stunting among children under-5 years in Nigeria: a multilevel analysis. BMC Pediatr.

[21] Chapagain RH, White MC, Graham SM (2023). A cross-sectional study evaluating the prevalence and predictors of malnutrition among children and adolescents visiting an urban academic hospital in Nepal. Public Health Nutr.

